# Unexpected and Deadly: Three Cases of Bone Marrow Necrosis

**DOI:** 10.7759/cureus.9565

**Published:** 2020-08-05

**Authors:** Abigail S Chan, Nirali V Marvania, Nicholas B Burley, Roberto Martinez

**Affiliations:** 1 Internal Medicine, Sinai Hospital of Baltimore, Baltimore, USA; 2 Hematology and Oncology, Sinai Hospital of Baltimore, Baltimore, USA

**Keywords:** bone marrow necrosis, alcohol abuse, prostate cancer, gastrointestinal cancer

## Abstract

Bone marrow necrosis (BMN) is a rare pathological diagnosis, with an incidence of 0.3% to 2%. It is most often associated with hematological malignancies and less commonly due to solid tumors, infections, medications, sickle cell disease, chemotherapy, radiotherapy, or idiopathic causes. We reviewed bone marrow biopsies performed in our institution from 2009 to 2019 and found three cases of BMN. Two cases were secondary to neoplastic causes, while the third one was possibly from alcohol abuse.

## Introduction

Characterized by the disruption of the reticular bone marrow pattern with preservation of cortical bone, bone marrow necrosis (BMN) is a rare pathological diagnosis that portends a poor prognosis. Although more commonly described during autopsies, 0.3% to 2% of cases are diagnosed antemortem [1,2]. It is most often associated with hematological malignancies and less commonly from metastatic solid tumors, infections, medications, sickle cell disease, chemotherapy, radiotherapy, or idiopathic causes [3,4].

There is scant knowledge about this pathological finding, and the purpose of this study is to add to the limited literature on BMN. We reviewed bone marrow biopsies read in our institution from 2009 to 2019 and found three cases of bone marrow necrosis, which are described herein.

## Case presentation

Case 1

A 63-year-old man with hypertension, subsegmental pulmonary embolism, and recent diagnosis of prostate cancer was admitted into our institution for several days of fatigue. He had a history of vitamin B12 deficiency, normochromic and normocytic anemia, with a baseline hemoglobin level of 12.5 mg/dL. In relation to his prostate cancer, he underwent decompressive laminectomy with adjuvant radiotherapy for a thoracic cord compression ­­­15 months prior and was started on leuprolide and denosumab the year before.

**Figure 1:**
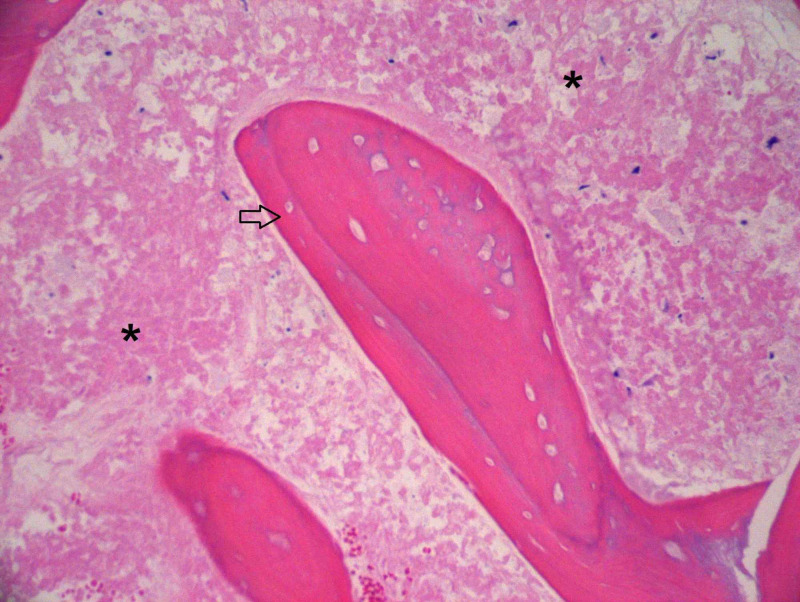
Hemotoxylin- and eosin-stained section of the bone marrow core biopsy showing necrosis of the bone, osteocytes (arrow), and the adjacent marrow with essentially absent hematopoietic elements (asterisk). 100x magnification.

His physical examination was significant for pale conjunctiva and an irregularly irregular heart rhythm from atrial fibrillation. Admitting complete blood count (CBC) showed hemoglobin of 5.4 mg/dL, hematocrit 17.1%, white blood cell (WBC) count 5.03 k/mm3, and platelet levels at 200 k/mm3. Peripheral smear showed anemia with nonspecific erythrocytic morphology. He received a transfusion of packed red blood cells and continued to require transfusions during his hospitalization. His bone marrow biopsy showed partial infarction with fibrosis. Hematopoietic elements were essentially absent. S100, cytokeratine, and CD45 were all negative. Reticulin and trichrome stains both showed moderate fibrosis. Iron stain showed a normal amount of stainable iron (Figure 1). His hospital course was complicated by progressive metastasis in T2 to S2 vertebrae requiring further radiotherapy and hypoxic respiratory failure leading to mechanical intubation. He later succumbed to his disease.

Case 2

A 71-year-old man with underlying hypertension, diabetes mellitus, psoriasis, and dyslipidemia came in for weight loss, progressive weakness, abdominal pain, and lower back pain. Due to his constellation of symptoms, he was unable to ambulate well. He lost approximately 20 pounds in the last month. The MRI of his thoracic and lumbar spine demonstrated diffuse osseous changes consistent with metastases, while the MRI of the brain did not show any lesions. Computerized tomography (CT) scans of the abdomen and pelvis revealed bilateral pleural effusions, ascites, and a small pericardial effusion. Upper and lower endoscopies revealed gastritis and a non-bleeding duodenal ulcer. An evaluation for prostate cancer was negative.

**Figure 2:**
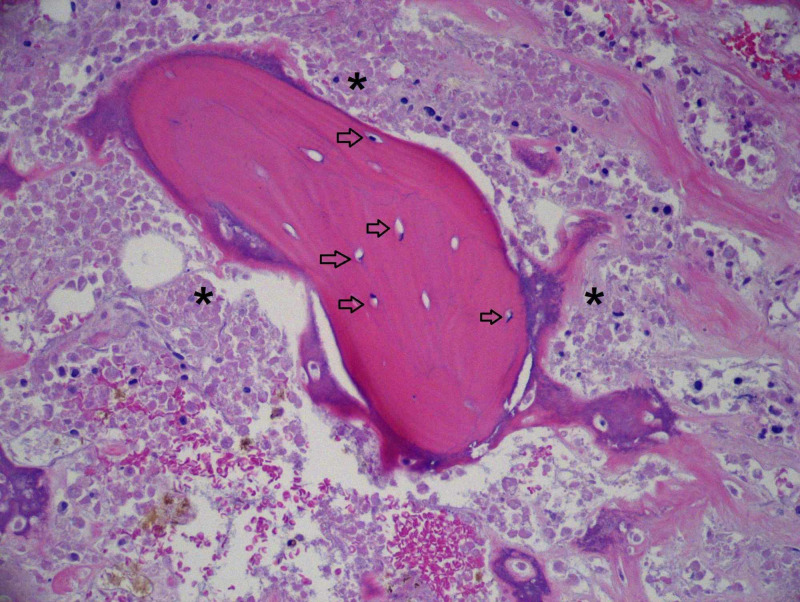
There is viable bone trabecula with dark osteocyte nuclei visible (arrows). The cells surrounding the trabecula are necrotic (asterisks). 100x magnification. Hemotoxylin- and eosin-stained section.

Laboratory findings on admission were significant for hemoglobin 13.8 mg/dL, hematocrit 43%, WBC count 5.49 k/mm3, platelets 118 k/mm3, sodium 131 mmol/L, blood urea nitrogen 24 mg/dL, alkaline phosphatase (ALP) 3,842 unit/L, and calcium 10.2 mg/dL. Multiple myeloma workup was negative. MRI of the left femur showed diffusely heterogenous marrow suspicious for osseous metastases. Given the osseous lesions and the thrombocytopenia, bone marrow biopsy was performed. Bone marrow necrosis was evident, but no definite tumor was identified with cytokeratin immunostain (Figure 2). Flow cytometry of biopsy showed dominance of mature granulocytes and lymphocytes suggesting significant peripheral blood component. A biopsy of left femur revealed adenocarcinoma (CK7/CK20/CDX2+) suggestive of pancreaticobiliary or gastrointestinal tract source. Multiple tumor markers were elevated including: CA-125 was 5,618, CEA was 627, and CA 19-9 was 38,400. Magnetic resonance cholangiopancreatography showed numerous hepatic lesions. He developed worsening pancytopenia, leading to neutropenic fever, polymicrobial bacteremia, and septic shock, eventually leading to his demise shortly after intensive care unit (ICU) admission.

Case 3

A 52-year-old woman with a history of hypertension, diabetes mellitus, heart failure, hypothyroidism, chronic pancreatitis, and treated hepatitis C was seen in our emergency department for left lower quadrant abdominal pain and vomiting for the past 2 days. She has a long history of alcohol abuse, consuming two to three bottles of wine daily and had previous admissions for alcohol withdrawal. She had tenderness on the left lower quadrant of her abdomen.

**Figure 3:**
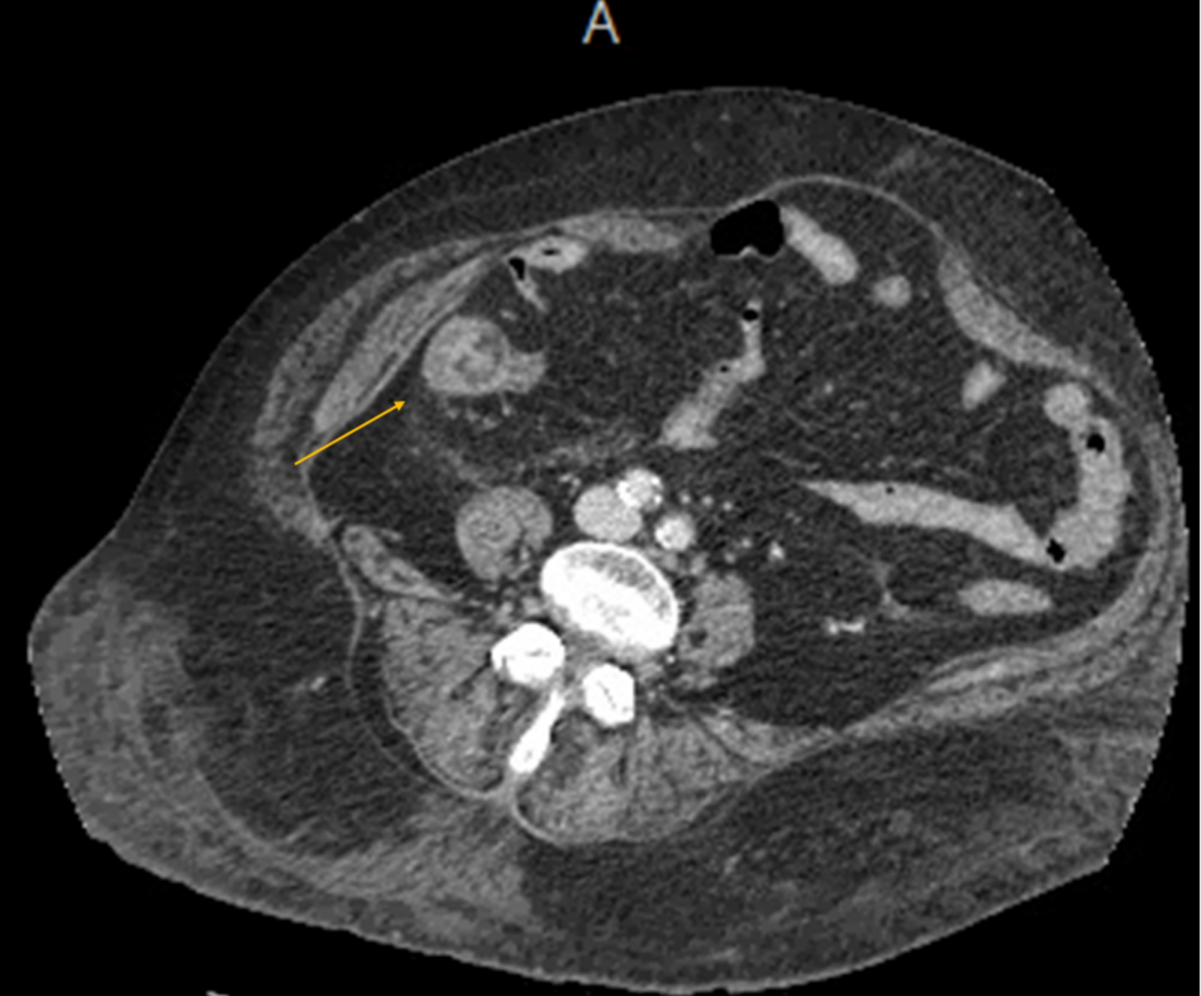
CT scan showing colitis, most prominent in the right colon (arrow).

Her CBC was significant for hemoglobin of 3.3 mg/dL, hematocrit 10.2%, WBC 4.03 k/mm3, and platelet levels 4 k/mm3. The corrected absolute reticulocyte count was 0.9. The peripheral smear showed macrocytic anemia with target cells and thrombocytopenia with no evidence of schistocytosis, stelliosis, or aggregation. The lactate dehydrogenase (LDH) levels were 407 unit/L, and ALP was 89 unit/L. The alcohol level on admission was undetectable, <10 mg/dL. Liver function tests were aspartate aminotransferase (AST) 96 unit/L, alanine transaminase (ALT) 48 unit/L, and international normalized ratio (INR) was elevated, 2.2. CT of the abdomen was suggestive of colitis, most prominent in the right colon (Figure 3).

**Figure 4:**
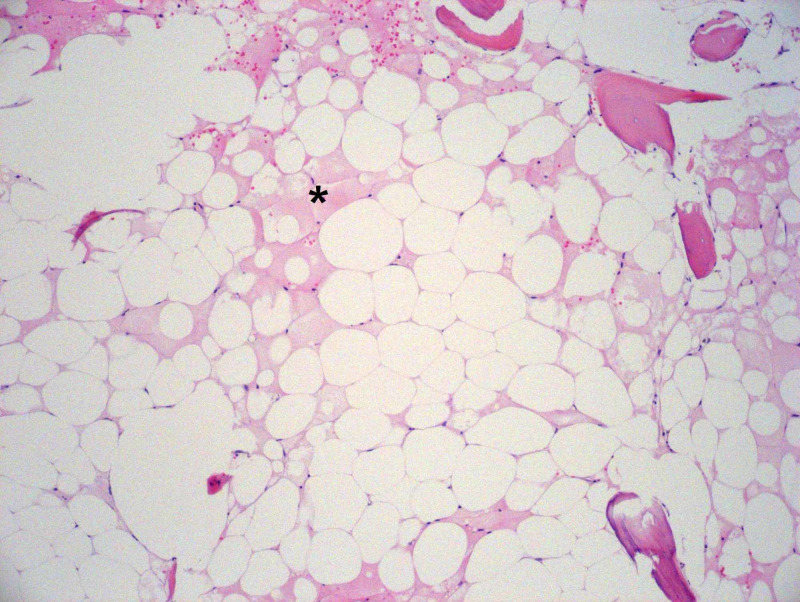
Hemotoxylin- and eosin-stained section of the bone marrow core biopsy showing necrosis of marrow space (asterisk). 100x magnification.

During her hospital stay, she continued to have pancytopenia. The bone marrow biopsy revealed predominantly infarcted marrow space (Figure 4). The residual marrow was normocellular with adequate erythropoiesis and megakaryopoiesis. No erythropoietic dysplasia or excess blasts were noted. Myelopoiesis was diminished with scant but maturing myeloid elements. Abundant stainable iron and mild reticulin fibrosis were found. No evidence of infection or neoplasia. Clinically, she had no evident source of bleeding. She was discharged with stable hematogical parameters and advised close follow-up and alcohol cessation. Shortly after discharge, she was admitted after an episode of cardiac arrest that was followed by a complicated intensive care unit stay, resulting in transition to hospice. 

## Discussion

Looking into the 10-year history of a large academic community hospital, we were only able to identify three cases of BMN. Of those, two were attributed to neoplastic causes. The third one was of unclear cause, but possibly from “marrow toxicity” from the patients multiple medical issues or from alcohol.

BMN was initially described in a patient with sickle cell disease in 1941 [5]. It presents as a localized or diffuse process, causing destruction of the hematopoietic tissues with preservation of the cortical bone [1]. Its pathophysiology remains unclear. Several mechanisms have been proposed: (1) bone marrow infiltration with neoplastic processes; (2) sinusoidal obstruction syndrome related to endothelial injury from cytotoxic agents, endotoxin exposure, radiation therapy, or sickling erythrocytes; and (3) cytokine release by macrophages and monocytes causing downstream systemic inflammatory effects and platelet aggregation, leading to vascular disruption, cell hypoxia, and eventually necrosis [1,4,6-7]. Our first two cases were associated with extensive bony metastases, and our third case was associated with multiple medical problems including alcohol abuse and alcohol withdrawal.

In a previous series of BMN, hematological malignancies predominated (60%) specifically acute lymphocytic leukemias (ALL), followed by metastatic solid tumors and infectious causes. The solid tumors most commonly associated with BMN were gastric cancers, prostate cancers, Ewings sarcoma or primitive neuroectodermal tumors (PNETs), neuroblastoma, colon cancers, and lung cancers (3,6). The first two cases were due to metastatic prostate and gastrointestinal cancers. Nonmalignant causes include infections (i.e., Q fever, mycobacterium, human immunodeficiency virus, parvovirus, mucor), drugs (i.e., sulfasalazine, granulocyte-colony-stimulating factors, fludarabine, interferon-alpha, all-trans retinoic acid), sickle cell disease, hyperparathyroidism, anorexia nervosa, hemolytic uyremic syndrome, antiphospholipid syndrome, disseminated intravascular coagulation, and idiopathic causes [1].

Typically, patients were present with bone pain, fatigue, and fever. This was true for cases 2 and 3. Laboratory findings usually reveal abnormal hemograms and elevated markers of cellular necrosis, such as LDH, ALP, uric acid, and transaminases. Anemia, thrombocytopenia, and leukoerythroblastic reactions can be found [1,6,8]. For our patients, a summary of their laboratory results can be found in Table 1. Performing a bone marrow biopsy is definitive, showing amorphous and eosinophilic material dispersed in extracellular spaces, rare cells with poorly delimited cytoplasmic membranes, irregular nuclei, and shrunken cytoplasm. BMN is further classified as grade I, grade II, or grade III corresponding to <20%, 20%-50%, or >50% bone marrow involvement, respectively [6,9]. Interestingly, prognosis does not seem to correlate with the grade of BM involvement [10]. Perhaps, this is because prognosis is more directly related to the patient’s underlying medical conditions.

**Table 1:** Description of symptoms and laboratory values present in each case M: male; F: female; GI: gastrointestinal; WBC: white blood cells (K/mm3); Hgb: hemoglobin (g/dL); Hct: hematocrit (%); Plt: platelet (K/mm3); LDH: lactate dehydrogenase (unit/L); ALP: alkaline phosphatase (unit/L).

Case #	Age	Sex	Underlying disease	Presenting symptom	Associated symptoms		Hematological parameters				Chemical parameters		Necrosis on biopsy	Biopsy to death (days)
					Fever	Bone pain	WBC	Hgb	Hct	Plt	LDH	ALP		
1	63	M	Metastatic prostate cancer	Fatigue	Negative	Negative	5.03	5.4	17.1	200	542	376	Partial	80
2	71	M	Metastatic cancer of GI origin	Weakness and low back pain	Negative	Positive	5.49	13.8	43.0	118	None	3842	Partial	23
3	52	F	Heavy alcohol use	Left lower abdominal pain and vomiting	Negative	Negative	4.03	3.3	10.2	4	407	89	Predominant	85

The approach to BMN involves the treatment of the primary etiology, supportive care, and blood transfusions. Malignancy-related BMN mortality is as high as 55% [3]. Reversibility of BMN was found in some patients with underlying ALL, neuroblastoma, hairy cell leukemia, and one with an idiopathic cause [11-14]. Bone marrow aplasia secondary to heavy alcohol use is potentially reversible. Unfortunately, our patient had infarction and fibrosis at the time of presentation, representing a later stage of marrow damage [15]. All of our cases died shortly after their diagnosis, with an average of 62.7 days since the bone marrow biopsy. It is difficult to ascertain whether our patients succumbed to the severity of their multiple comorbidities, progression of their malignancy, the sequelae of pancytopenia, or a combination of these factors, but pancytopenia was a prominent finding and complication in the course of their illness.

## Conclusions

Underlying causes for BMN are numerous and difficult to identify contributing to the underestimation of cases. Prognostic factors are not well-defined, but those with malignancies/high tumor burden are associated with higher mortality. A high level of suspicion for BMN is warranted in cases of pancytopenia with high levels of LDH and ALP. This is especially true for cases wherein an inciting factor such as chemotherapy, radiation therapy, or toxin exposure is present. Some cases may be reversible, thus early detection is important to potentially improve outcomes.
